# Eight quick tips for requesting materials from natural history collections

**DOI:** 10.1371/journal.pcbi.1014753

**Published:** 2026-09-10

**Authors:** Giovanna Yumi Scorsim Omura, Denise Furr, Denis Jacob Machado

**Affiliations:** 1 Department of Bioinformatics and Genomics, College of Computing and Informatics, University of North Carolina at Charlotte, Charlotte, North Carolina, United States of America; 2 Center for Computational Intelligence to Predict Health and Environmental Risks (CIPHER), University of North Carolina at Charlotte, Charlotte, North Carolina, United States of America; 3 Schiele Museum of Natural History, Gastonia, North Carolina, United States of America; Montreal, CANADA

## Introduction

Natural history collections (NHCs) hold preserved specimens and artifacts from the natural world. They function as a large archive of biodiversity and geological history. These collections help address many current challenges, including climate change, public health, and resource management [1]. The principles for effective collaboration discussed here can also be useful in other scientific partnerships beyond NHCs.

A recent study found that the 73 largest NHCs in 28 countries hold over 1.1 billion objects [2]. These include zoological collections and herbaria. This huge number shows the scientific value of these collections, which range from dinosaur fossils to tiny pollen grains. Estimates suggest there may be nearly three billion specimens worldwide, but it is hard to count them all because of differences in how sub-collections are described and ongoing digitization [3].

Digitization and online databases have made collections much easier to access. Still, obtaining physical specimens for research often means navigating complex procedures and varying loan policies at each institution. There are no standard steps for making effective specimen loan requests. This lack of standardization can slow down researchers who need samples for things like DNA extraction, morphological analysis, or high-resolution imaging.

**Fig 1:**
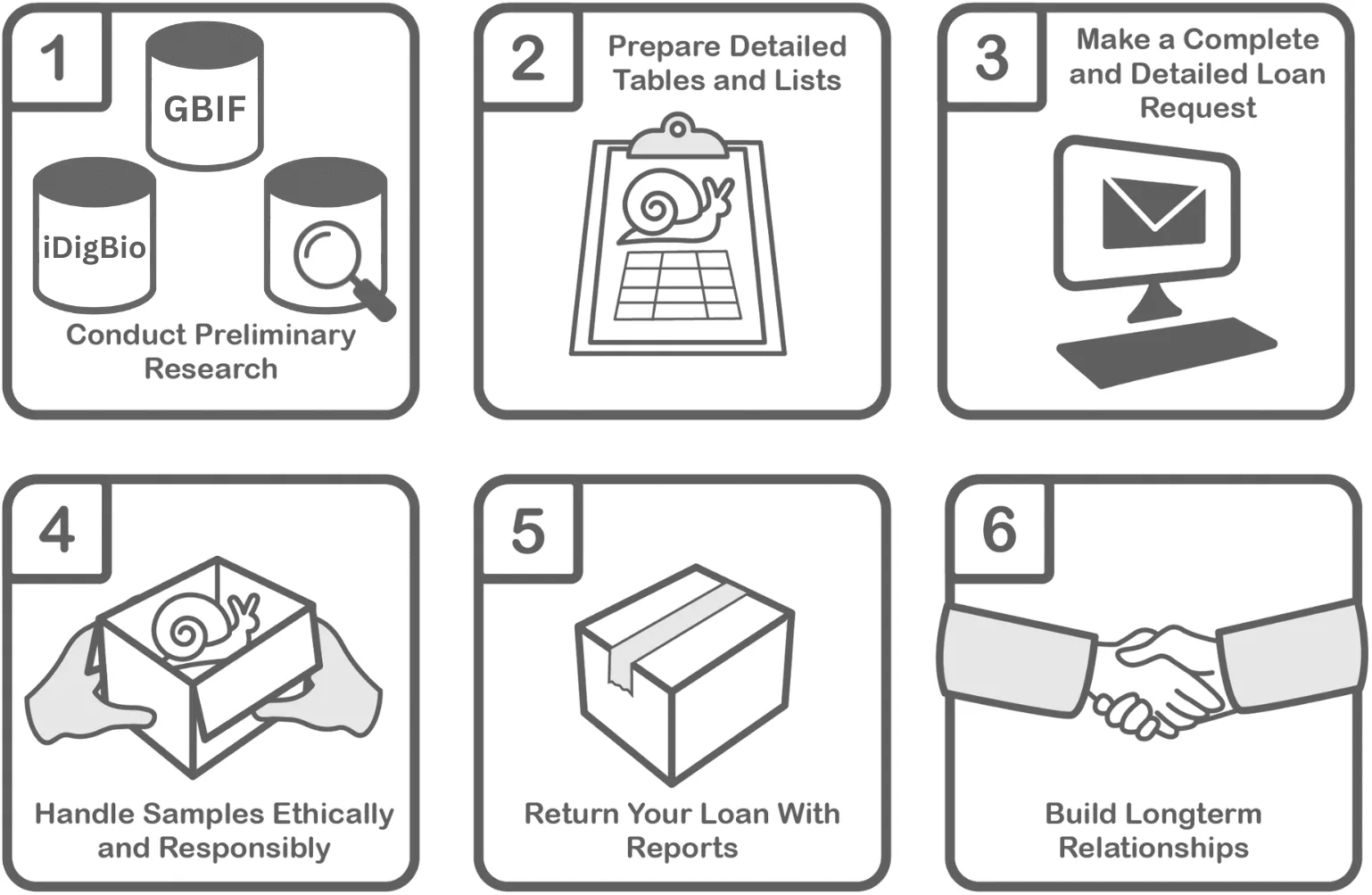
Our guide provides researchers with best practices for navigating the specimen loan lifecycle, from preliminary research and material selection to sample handling and reporting, to foster enduring, collaborative relationships with biorepositories and natural history collections.

We offer eight quick tips to facilitate NHC loan requests. These suggestions come from our experience and research with zoological collections, especially mollusks. Our aim is to help researchers access valuable resources while protecting specimen integrity and long-term preservation. We also want to build good relationships with curators around the world. Fig 1 shows the full workflow, and a detailed step-by-step checklist is available in S2 Text.

## Tip 1: Understand the databases available to you

NHCs often have collection management systems (CMS) and publish their specimen information to data aggregators that harvest records from different collections (see Table 1). Learn how to use these databases effectively to query potential specimens for your study. Filter searches by species, location, date, preparation type (e.g., skull, skin, fluid-preserved, tissue) and other relevant criteria. Note that data aggregators may have incomplete or missing information compared to the primary collection management systems. This is because the CMS often has information that is not harvested and because some records may be restricted for public access (e.g., endangered species localities). The collection CMS also may have more current information because data updates (e.g., taxonomy, locality, date) may not be refreshed in the data aggregator.

**Table 1:** Examples of online databases with information from different NHCs.

Name	Type	Website
Arctos [4]	CMS	https://arctosdb.org/
Specify Web Portal [5]	CMS	https://www.specifysoftware.org/
Symbiota [6]	CMS	https://symbiota.org/
Global Biodiversity Information Facility (GBIF) [7]	Aggregator	https://www.gbif.org/
Integrated Digitized Biocollections (iDigBio) [8]	Aggregator	https://idigbio.gbif.us/
VertNet [9]	Aggregator	https://www.vertnet.org/

Familiarize yourself with database search operators and wildcard usage. Understand the data fields most relevant to your research. Many of these databases offer workshops, tutorials, and other learning resources. Do not hesitate to use them. This step helps you determine whether the specimens you need exist and where they are located. It also helps to interactively refine the initial research question based on the results of these searches.

## Tip 2: Prepare a meticulous and accurate specimen list

Before proceeding, ensure that your specimen list is accurate and complete. Utilize taxonomic resources such as field guides, monographs, and online databases to verify identifications. Incorrect identification can lead to wasted time and resources for both you and the collection staff. If you are uncertain about an identification, consult a taxonomic expert. Explain why you need these specimens, including detailed information such as their scientific names, localities, collectors, collection catalog numbers, preparation types, and dates. This helps the curator locate the exact specimen you need. If providing specific catalog numbers is not possible, you can also describe basic information such as sex, condition, and quantity.

Include your personal information: your contact details, the name of your institution, a specimen list, and the person (advisor, curator, staff) who has a permanent position at the institution receiving the material and will take responsibility for ensuring that it is handled without damage and returned to the museum in a timely manner. A well-prepared list demonstrates professionalism and increases the likelihood of approval. Presenting your request in a structured format improves clarity and facilitates verification by curators. A partial example of the desired specimens, formatted for submission, is shown in Table 2.

**Table 2:** Example of how to organize desired specimen records from a museum collection. The table includes taxonomic identity, locality, and sampling requirements. The column “Specimen Count” represents the total number of individual samples or specimens preserved together within that specific cataloged lot. The column “Individuals Needed” indicates the exact number of individual samples or specimens from that lot required to meet the operational demands of our research design.

Museum Code	Catalog No.	Genus	Species	Specimen Count	Individuals Needed	State	Preparation Type
FLMNH	284376	*Triodopsis*	*anteridon*	1	1	TN	Tissue
FLMNH	403232	*Triodopsis*	*claibornensis*	1	1	TN	Shell
FLMNH	504646	*Triodopsis*	*vannostrandi*	11	2	GA	Tissue
FLMNH	450342	*Triodopsis*	*vultuosa*	9	2	TX	Tissue

## Tip 3: Craft an effective loan request

Before you send the specimen list, describe your research project in detail. Share your research questions, methods, and what you hope to find. Explain why you need these specimens and how they will help your research, while assuring that your institution has the facilities and funds (if applicable) to complete your project. This information helps the curator see the value of your research and support your loan request. Mention your experience with similar specimens, methods proposed and include a timeline for your project. Be clear about how you will acknowledge the NHC in your publications and cite the individual specimens. Keep in mind: loans are typically made to advisors or permanent staff, not to students or postdoctoral associates; often no more than half of any taxonomic or locality series is usually loaned at one time; endangered or rare species likewise may be shipped in installments; loans may not be transferred to third parties or other institutions without prior approval; loans may not be used for a different project without prior written authorization; invasive procedures must be pre-approved; institutions may require copies of all derived media such as images and CT scans; and some loans may require permits to ship or transport.

State a clear scientific question and explain why your chosen methods and samples are necessary to answer it. Try to use less invasive or destructive sampling methods when possible to get reliable molecular data, making your best effort to preserve morphological diagnostic features. In the case of historical material usage, include a detailed justification for why you need to destructively sample those particular specimens of historical importance [10]. High-throughput sequencing can provide useful data from small tissue samples [11,12]. Make sure you understand the museum’s loan policies before you submit your request, as each museum has its own guidelines. Shipment and permits are complex and require in-depth knowledge, often in consultation with collection staff. To understand key points of those rules you can use the Society for the Preservation of Natural History Collections (SPNHC) website (from https://spnhc.org/permitting/, go to Home → Resources → Permitting). To stay organized, create a folder for each museum and its communications. You can find a sample template letter for specimen loan requests in S1 Text.

## Tip 4: Handle specimens ethically and prioritize non-destructive methods

Follow the NHC’s rules for handling, storing, and analyzing specimens [13]. Use the right equipment and techniques to avoid causing damage. Keeping specimens in good condition is important for future research [14,15]. Learn about the correct storage conditions, such as temperature and humidity. Remember, specimens can be damaged by pests, mold, light, and changes in temperature, even when you are taking care of them.

If you must use destructive sampling, clearly explain why it is necessary [16]. Plan your sampling carefully to limit the impact on the collection and keep detailed records of what you do. In exceptional cases where a researcher is responsible for performing destructive sampling on a loaned specimen, care must be taken to preserve its taxonomically relevant structures.

When your materials arrive, let the collection managers know and check that everything is in good condition. Take photos or videos as you open the package. Send this documentation along with your signed invoice by email or mail. Before returning the signed invoice, annotate it with any condition issues and note if there are errors in the list of specimens provided (e.g., incorrect catalog numbers, additional or missing specimens on the invoice, etc.).

## Tip 5: Anticipate challenges and remain adaptable

Be prepared for potential challenges such as processing delays or restrictions on certain specimens. Processing delays may be attributed to staffing shortages, a backlog of requests being processed by the collection, or other factors (e.g., time required to get necessary permits, shipping logistics). Another challenge may involve finding the specimens of interest: while digitization enhances accessibility, records can be difficult to search if the data are not structured and controlled. Research often requires adjustments. Have a backup plan in case your first request is denied, and be ready to change your research design if needed.

Some specimens may be available in limited numbers or considered rare. Be aware that the collections staff may deny your request or choose to split it into different shipments so that not all specimens of a species from a locality are sent at once. Database records may also contain outdated taxonomic names and different systems may use different taxonomies, which can make it challenging to search records. Importantly, it is crucial to inform the collection if your research reveals a misidentified specimen. If funding is available and the specimen request volume is high and/or involves type, rare, or endangered material, consider an in-person visit to conduct the investigation.

## Tip 6: Communicate promptly throughout the loan period

Maintain regular communication with the curator throughout the loan period. Provide updates on your research progress and any findings. This builds trust and strengthens your relationship with the NHC. Respond promptly to any inquiries from the curator. Provide a thorough final report summarizing your research findings. Letting the collection know about publications that use their material is essential for maintaining a productive collaboration. If molecular sequences (DNA, RNA, or amino acids), CT scans, or isotope data are generated, submit results to public repositories (e.g., GenBank, Sequence Read Archive, Morphosource, IsoBank). Include the catalog numbers of voucher specimens in all submissions and publications. Any publication using materials from the NHC should adhere to the FAIR guiding principles for scientific data management, ensuring that research objects are findable, accessible, interoperable, and reusable.

## Tip 7: Return specimens on time and in proper condition

After completing your research, return specimens promptly and in the same condition in which they were received. You should reuse the same packaging materials that were used to ship the samples to you. This ensures that the specimens are available for future research by others. Follow the NHC’s guidelines for packing and shipping specimens. Provide a detailed inventory of the returned specimens. If the loan return involves an international shipment, make sure that you follow all permit requirements for returning the specimens by communicating with the NHC prior to shipment.

Document the return process by taking photos of the specimens as you pack them. This helps the curator check their condition and see any changes from your research. If you have extra DNA from your extraction, let the curator know and ask if they want it returned to the collection. Also, work directly with collections staff on whether any new samples you collected could be added to the collection. Donating new specimens is a great way to support and improve the collection [15]. In addition, provide any taxonomic updates, and ideally any data taken (measurements, character scoring), analyses performed (isotopes, genetic sequencing), or images generated (photos, CT scans).

## Tip 8: Build long-term relationships with curators and societies

Build relationships with curators and other researchers at NHCs. Go to conferences and workshops, and take part in collaborative projects. Good relationships help you access collections over the long term and encourage scientific teamwork [14]. Remember to thank the NHC in your publications, offer to help with collection maintenance or other tasks, and join scientific societies that are relevant to your work.

Making these connections is valuable at any stage of your career (see S3 Text for more networking tips [17]). Reach out to professors, advisors, or colleagues who already know curators or society members. They can introduce you and offer advice on requesting samples. Attending conferences, workshops, and symposia is a great way to meet curators in person. Let the museum know you are willing to share your research findings to help the wider scientific community. If your interests match the curator’s, suggest working together or co-authoring publications.

## Conclusion

Natural history collections are essential for learning about our planet’s history and future. To get the most out of these collections, it’s important to access them responsibly. These quick tips aim to make the specimen loan process easier and help researchers and collection managers work together. When researchers know how to use databases, and make clear requests, they set themselves up for successful loans.

Ethics are very important. Following the rules for handling, storing, and analyzing specimens helps protect them for the future. If you plan ahead, communicate clearly, and return specimens on time, you build trust and make future collaborations easier.

Forming lasting relationships with curators and scientific societies is key to keeping access to natural history collections sustainable. Working together on projects, giving credit, and sharing your research all help these collections grow and stay protected. We hope these tips help researchers from all fields use collections responsibly and support new scientific discoveries.

## Supporting information

S1 Text Template loan request letter. (PDF)

S2 Text Specimen loan process checklist. (PDF)

S3 Text Understanding challenges facing natural history collections and building professional networks. (PDF)
