# Supplementary material for: Eight quick tips for requesting materials from natural history collections
Source: PLoS Comput Biol. 2026 Sep 10;22(9):e1014753. doi: 10.1371/journal.pcbi.1014753 (PMC13561314; doi:10.1371/journal.pcbi.1014753)
Supplement: Supplementary file 1 [file pcbi.1014753.s001.pdf]

S1 Text. Template loan request letter

The following template can be adapted for requesting specimen loans from natural history collections. Replace bracketed placeholders with your specific information. Some sections are optional modular blocks, and it is left to the researcher to select the important paragraphs to use in your request.

Dear Curator [Curator's Name],

I am [your position and institution]. Please consider my request for the materials listed in the attached table.

1) Requested material and amount:
These materials are vital for executing [the project name and team involved]. I have also attached a lab report, which contains our justification for using tissue, specimens and volume for the success of [the analyses to be performed].

2) Expertise in caring for your samples:
We will do our very best to manage the materials responsibly and carefully, returning them to your collection as soon as possible and following your loan policy to the letter. We will let your collection know in case any publication uses information from the material you share with us. We will also share any and all results with you as soon as our analyses are completed.
Our plan is to use methods, such as [photography, morphometric analysis, DNA extractions, etc].

3) Sharing results:
If DNA/RNA sequences are analyzed based on your material, the results shall be submitted to public repositories such as those managed by NCBI (e.g., GenBank and SRA), and the catalog numbers of the voucher specimens will be cited. Finally, any publication using the materials we received from you will adhere to the F.A.I.R. guiding principles, ensuring that all research objects are findable, accessible, interoperable, and reusable for both machines and people.

4) Caring for left-over material:
It is possible that total DNA extraction will yield more material than we need. In that case, we will notify you, and any unused samples or extracted DNA will be returned to your collection (or held in secure storage and returned upon your request). We will ensure that information about any extracted DNA is made available to you and cited in any publication that utilizes these samples. We will not use samples for any other project nor transfer them to a third party without prior written authorization.

5) Shipping:
We are requesting the materials to be shipped to my laboratory at this address: [the complete address]. I will be happy to cover any shipping costs associated with this request.

Thank you for your time and consideration. I look forward to the possibility of collaborating with your institution.

Sincerely,
[Your Name]
[Your Affiliation]
[Your Contact Information]
