# Supplementary material for: Eight quick tips for requesting materials from natural history collections
Source: PLoS Comput Biol. 2026 Sep 10;22(9):e1014753. doi: 10.1371/journal.pcbi.1014753 (PMC13561314; doi:10.1371/journal.pcbi.1014753)
Supplement: Supplementary file 2 [file pcbi.1014753.s002.pdf]

S2 Text. Specimen loan process checklist

This checklist (divided into 6 phases that are summarized in Fig A) consolidates the actionable steps involved in requesting, handling, and returning specimens from natural history collections (NHCs). It is organized by phase to help researchers track their progress throughout the loan process.

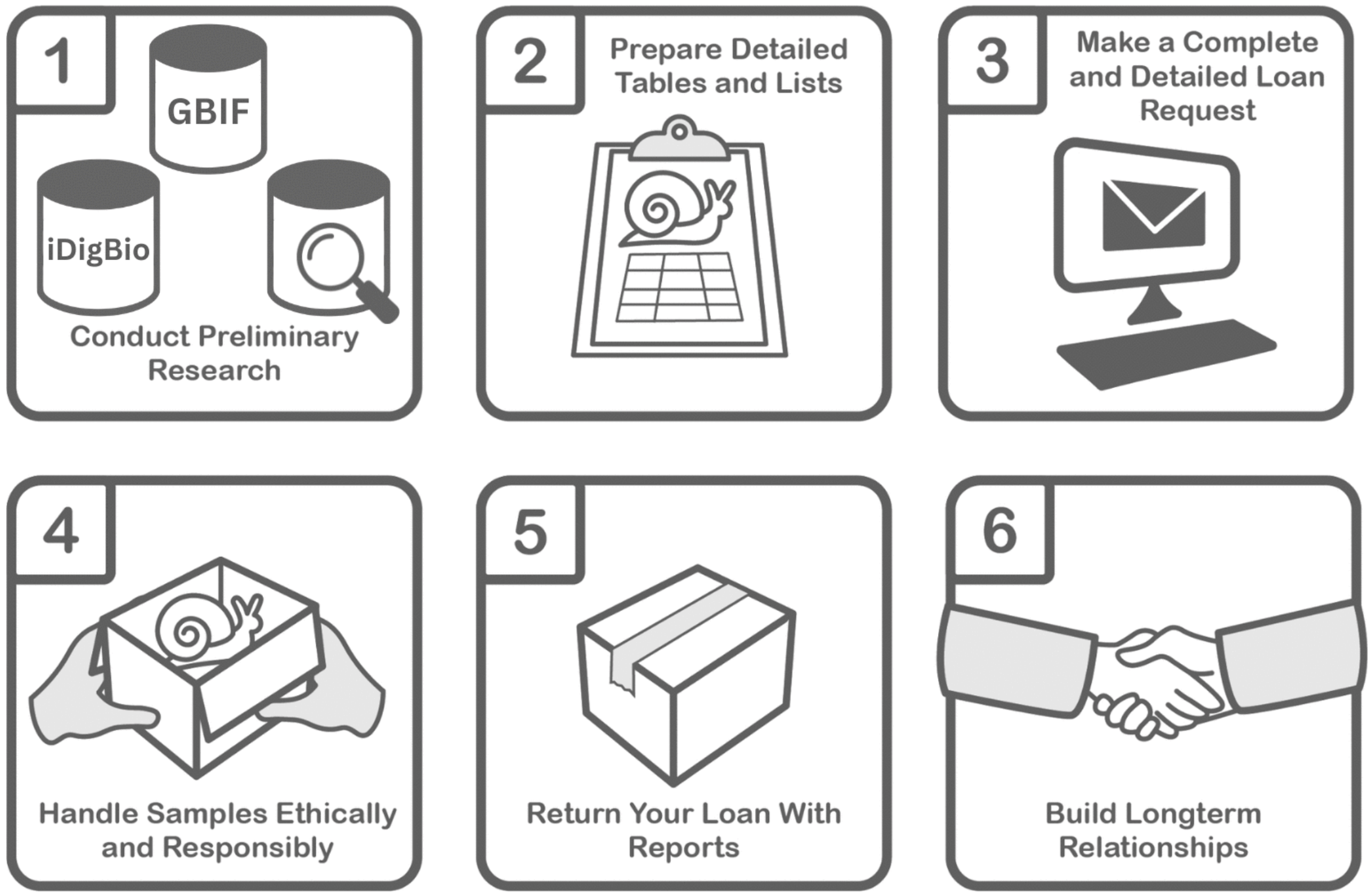

Fig A. The six phases of the species loan process. Phase 1: conduct preliminary research. Phase 2: prepare detailed tables and lists. Phase 3: make a complete and detailed loan request. Phase 4: handle samples ethically and responsibly. Phase 5: return your loan with reports. Phase 6: build long term relationships.

Phase 1: Conduct preliminary research

1. Search online databases (e.g., Arctos, GBIF, iDigBio, Specify, or the NHC's own database) to identify available specimens.
2. Learn how to use database search operators, wildcards, and relevant data fields.
3. Use database workshops, tutorials, and other learning resources offered by these platforms.
4. Refine your research question based on specimen availability.
5. Verify all taxonomic identifications using field guides, monographs, and online resources.
6. Consult a taxonomic expert if you are uncertain about any identification.

Phase 2: Prepare detailed tables and lists

7. Prepare a detailed specimen list with catalog numbers, scientific names, localities, collectors, preparation types, and the number of individuals needed.
8. Format the specimen list as a clear spreadsheet or table for submission.

9. Create a dedicated folder for each museum and its associated inquiries.
10. Review the museum's collection policies and procedures before submitting your request.

Phase 3: Make a complete and detailed loan request

11. Write a cover letter that includes a description of your research project, research questions, methods, and expected outcomes (see S1 Appendix for a template).
12. Separate out destructive/consumptive/invasive sampling requests as a separate category of loan from non-destructive specimen requests.
13. In your project make sure to add qualifications/previous experience of the researcher in proposed sampling methods, sample volume, a list of other institutions contacted to ensure that no single NHC receives the full burden of consumptively sampling their holdings, and that you will meet NHC loan requirements, such as posting results in a publicly accessible database like NCBI.
14. Include a statement of your qualifications and experience in handling specimens.
15. Provide a research timeline.
16. Justify why the requested specimens are necessary and sufficient to address your research question.
17. Describe whether non-destructive or destructive methods will be used and justify the choice. If destructive sampling is necessary, provide a detailed written justification for why that method is necessary to meet the project goals.
18. Explain how you will acknowledge the NHC and cite specimens in your publications.
19. State your willingness to cover shipping costs.
20. Ensure that all permits and other required shipping documents are in order prior to shipment (especially for international loans)
21. Attach the formatted specimen list to your request.

Phase 4: Handle samples ethically and responsibly

22. Inform the collection managers upon receipt of the shipment.
23. Take photographs or video while opening the package to document the condition of specimens upon arrival.
24. Verify that all specimens match the invoice and are intact. Take notes at the invoice in case of condition issues (i.e., condition of a specimen is different from what's on the invoice), and if there are any errors in the list of specimens provided.
25. Sign and return the invoice by email or mail along with the photographic documentation.
26. If anything is damaged during transportation, report it immediately with photographic evidence.
27. Follow the NHC's guidelines for handling, photographing, locked storage, and recording data from specimens.
28. Store specimens under appropriate conditions (temperature, humidity) as specified by the lending institution.
29. Document all sampling procedures thoroughly.

During the loan period

30. Maintain regular communication with the curator.
31. Provide updates on research progress and preliminary findings.
32. Respond promptly to any inquiries from the curator.
33. Adhere to FAIR guiding principles for data management in all publications and data submissions, citing properly formatted catalog numbers for the specimens used.
34. Notify the curator about any publications that use information from the loaned material.

Phase 5: Return your loan with reports

35. Return specimens promptly after completing your research.
36. Ensure that all permits and other required shipping documents are in order prior to shipping the specimens back to the collection (especially for international loans).
37. Ensure specimens are in the same condition in which they were received.
38. Reuse the original packaging materials when possible.
39. Follow the NHC's guidelines for packing and shipping.
40. Prepare a detailed inventory of the returned specimens.
41. Take photographs of specimens during packaging to document their condition at return.
42. If DNA extraction produced surplus material, notify the curator and ask whether they prefer the extracted DNA returned to the collection.
43. Provide a final report summarizing your research findings to the curator.
44. Report any taxonomic misidentifications discovered during your research to the curator.
45. Submit DNA or RNA sequences, CT scans, isotope data, etc. to public repositories (e.g., GenBank, Sequence Read Archive, Morphosource, IsoBank) and include voucher specimen catalog numbers. Provide taxonomic or other updates, measurements, character scores, or photographs to the NHC.

Phase 6: Build longterm relationships

46. Share all published results with the lending institution.
47. Acknowledge the NHC and cite the specimens in all publications.
48. Consider whether newly collected samples from your research could contribute to the collection.
49. Maintain the relationship with the curator for future collaborations.
