# Supplementary material for: Eight quick tips for requesting materials from natural history collections
Source: PLoS Comput Biol. 2026 Sep 10;22(9):e1014753. doi: 10.1371/journal.pcbi.1014753 (PMC13561314; doi:10.1371/journal.pcbi.1014753)
Supplement: Supplementary file 3 [file pcbi.1014753.s003.pdf]

S3 Text. Understanding challenges facing natural history collections and building professional networks

This appendix provides expanded guidance on two topics that are summarized in the main text. The first section describes the operational challenges that natural history collections face. Understanding these challenges helps researchers anticipate potential obstacles and approach the loan process with informed expectations. The second section provides detailed networking advice for early-career researchers who are building relationships with collection managers and scientific societies.

Operational challenges facing natural history collections

Natural history museum collections face numerous challenges that can affect their ability to process and fulfill loan requests. Researchers who understand these challenges are better positioned to collaborate productively with collection staff.

According to the Society for the Preservation of Natural History Collections (SPNHC), museums and natural history repositories must carefully manage legal and compliance documentation for all specimen transactions, which includes tracking loans, borrows, gifts, and exchanges. When transporting, exporting, or re-importing specimens for loan transactions, institutions may need to obtain specific permits, such as a Transport Permit for moving regulated specimens across domestic borders, or specialized import and export permits if the transaction is international. Furthermore, for transactions involving protected species, institutions can utilize specialized international systems like the Certificate of Scientific Exchange (COSE). Under CITES regulations, a COSE authorizes the non-commercial loan, donation, or exchange of listed specimens between registered scientific institutions, ensuring that the movement of these research materials remains strictly compliant with international conservation laws.

The physical preservation of specimens is a constant concern. Specimens are vulnerable to damage from pests, mold, light, and temperature fluctuations [1]. Maintaining appropriate storage conditions requires continuous monitoring and resources that are not always available. These preservation demands compete with other institutional priorities for limited budgets.

Financial constraints and staffing shortages pose significant challenges to the operation of museums [2]. Budget cuts can limit resources for collection care, research, and public engagement. A lack of trained staff can hinder critical activities such as specimen cataloging, digitization, and loan processing. These limitations may result in longer response times for loan requests.

The digitization of collections presents both opportunities and challenges. While digitization enhances accessibility and enables large-scale data analysis, it also generates vast amounts of data that must be organized and managed effectively [3]. Not all specimens have been digitized. In many cases, only a fraction of the total holdings are represented in online databases. Researchers should be aware that the absence of a record in a database does not necessarily mean the specimen does not exist.

Human error is an inherent part of collection management. Database records may not always match the physical specimen. For example, a species name in the database may have changed over time due to taxonomic revisions, but the record was not updated accordingly. Researchers may only discover such discrepancies upon receiving the specimen and inspecting the identification tag. It is important to report these discrepancies to the curator so that the records can be corrected. Similarly, if research results indicate that a specimen should be assigned to a different species, this information should be communicated to the collection promptly.

Networking guidance for early-career researchers

Building strong relationships with natural history museum curators and scientific societies is invaluable for researchers at all career stages. For graduate students and early-career researchers in particular, these connections can provide access to unique resources, specialized expertise, and opportunities for collaboration [4].

Leveraging existing connections

Reach out to professors, advisors, or colleagues who may have existing connections with curators or society members. They can provide introductions and offer advice on how to approach sample requests. A personal introduction from a trusted colleague can make a significant difference in how a request is received.

Engaging at conferences and workshops

Participating in relevant conferences, workshops, and symposia is one of the most effective ways to meet curators and society members in person. Engage in discussions during sessions and poster presentations. Ask thoughtful questions that demonstrate your interest in the collection's holdings. Exchange contact information with the people you meet. These events offer excellent opportunities to initiate professional relationships in an informal setting.

Demonstrating collaborative intent

Express your willingness to share research findings with the museum. Contributing results to the broader scientific community demonstrates that you value the collection and respect the effort involved in maintaining it. If your research interests align with those of a curator, propose potential collaborations. Co-authorship on research papers or publications can be mutually beneficial and strengthen the partnership.

Contributing to collections

During your research, you may collect new samples on your own. Prior to conducting research, coordinate with local museums to see if newly collected samples can contribute to existing collections. Donating specimens is a meaningful way to build goodwill and enhance the collection with new material. Curators appreciate researchers who give back to the community that supports their work. Having discussions with NHCs prior to embarking on field work will result in the most synergistic partnership and may even guide the project design.

Joining scientific societies

Join relevant scientific societies that are connected to your taxonomic group or research area. Society memberships provide access to newsletters, mailing lists, and member directories that can help you identify potential collaborators and collection managers. Many societies also organize workshops specifically focused on collection-based research methods.

References

[1] Davis CL, Guralnick RP, Zipkin EF. Challenges and opportunities for using natural history collections to estimate insect population trends. Journal of Animal Ecology. 2023;92(2):237–249.

[2] Rohwer VG, Rohwer Y, Dillman CB. Declining growth of natural history collections fails future generations. PLoS Biology. 2022;20(4):e3001613.

[3] Macklin J, Shorthouse D, Glöckler F. I Know Something You Don't Know: The annotation saga continues.... Biodiversity Information Science and Standards. 2023;7:e112715.

[4] Byrne AQ. Reimagining the future of natural history museums with compassionate collection. PLoS biology. 2023;21(5):e3002101.
